# Identification of Yeast and Yeast-Like Fungi at a Tertiary Care Center: A Retrospective Study

**DOI:** 10.7759/cureus.62680

**Published:** 2024-06-19

**Authors:** Yogendra P Shelke, Ashwini A Tidake, Sarita Ugemuge, Prasanna Nakate, Suvarna Patil, Shailesh Lambhore, Nandkishor J Bankar, Gulshan R Bandre

**Affiliations:** 1 Microbiology, Bhaktshreshtha Kamalakarpant Laxmanrao Walawalkar Rural Medical College, Chiplun, IND; 2 Microbiology, Datta Meghe Medical College, Datta Meghe Institute of Higher Education and Research, Wardha, IND; 3 Microbiology, Shri Vithalrao Joshi Charities Trust’s College of Advanced Studies, Chiplun, IND; 4 Microbiology, Jawaharlal Nehru Medical College, Datta Meghe Institute of Higher Education and Research, Wardha, IND

**Keywords:** yeast-like fungi, antifungal susceptibility, non-albicans candida, c. albicans, yeast

## Abstract

Background

Fungal infections pose a significant global health challenge. Despite their substantial impact, these ubiquitous fungi can become pathogenic but have not received adequate attention in public health, leading to infections that are often underestimated by the general public and healthcare professionals. *Candida *species and *Cryptococcus *species play a key role in these infections, with emerging multidrug resistance in *Candida *species posing considerable challenges. This study aimed to understand the prevalence of yeast and yeast-like infections, particularly in the COVID-19 era, and to assess the antifungal susceptibility pattern.

Methodology

A retrospective observational study was conducted at a rural tertiary care medical college in Maharashtra, India. Retrospective records of samples processed for fungal culture were analyzed in the microbiology department. Yeast identification and antifungal susceptibility were performed using the VITEK-2 automated system.

Results

Among 95 fungal isolates, 86 (90.52%) were yeast isolates, primarily non-albicans* Candida* (NAC) species. *Candida albicans* accounted for 41 (47.67%) yeast isolates. In 14 isolates, NAC species were not identified by the VITEK-2 system up to the species level. Isolates from urine samples contributed the highest percentage of 61% (58) of yeast isolates. *C. albicans* showed high sensitivity to most antifungal agents. Other *Candida species*, such as *Candida famata, Candida parapsilosis*, and *Candida guilliermondii*, were sensitive to all antifungal agents. *Candida auris* showed complete resistance to amphotericin B and fluconazole but sensitivity to other agents. Mixed sensitivity patterns were observed in *Candida ciferri* and *Candida lusitaniae*, with some resistance to voriconazole, caspofungin, and micafungin.

Conclusions

This study shows the increasing prevalence of yeast and yeast-like infections, particularly NAC, during the COVID-19 era. Improved yeast identification and susceptibility testing are crucial for guiding the appropriate treatment and mitigating the impact of these infections, emphasizing the need for comprehensive future studies in this area.

## Introduction

Fungal infections pose a significant global health challenge, affecting approximately 150 million people annually and resulting in 1.7 million deaths [1]. Despite their substantial impact, fungal diseases, which are ubiquitous in the environment and can become pathogenic under specific conditions, have not received adequate attention in public health, leading to infections that are often underestimated by the general public and healthcare professionals [1,2]. Yeast is a unicellular fungus, with the pathogenic yeast genera *Candida* and *Cryptococcus* being the most common [3]. *Candida* species are responsible for approximately 90% of yeast infections worldwide [4]. The emergence of *Candida auris*, a multidrug-resistant yeast species discovered in 2009, highlights the growing threat posed by fungal pathogens, particularly in healthcare settings. *C. auris* is highly transmissible, capable of surviving on surfaces for >3 weeks, and is associated with severe infections and poor clinical outcomes [5,6].

Yeast infections have undergone significant changes, with non-albicans *Candida* (NAC) species now surpassing *C. albicans* as the main cause of candidiasis, particularly in the Asian continent [7,8]. *Candida tropicalis*, *Candida glabrata*, and *Candida krusei* are among the emerging NAC species driving this shift. In India, notable outbreaks have been linked to other yeast species such as *Pichia anomala*,* Pichia fabianii*, and *Kodamaea ohmeri *[9]. Furthermore, during the COVID-19 pandemic, fungal co-infections, especially involving *Candida* and *Mucor*, have become prevalent among affected individuals and are potentially exacerbated by the use of immunosuppressive therapies [10]. The objective of this retrospective observational study was to determine the prevalence of yeast and yeast-like infections, particularly in the era of COVID-19, and to assess the pattern of antifungal susceptibility.

## Materials and methods

Study design and setting

This retrospective observational study was conducted to identify and analyze yeast and yeast-like isolates in a tertiary care rural medical college and hospital in Dervan, Maharashtra. All yeast and yeast-like isolates from January 2018 to December 2021 were included in the study, which involved the analysis of various clinical samples, their species types, and susceptibility. The Strengthening the Reporting of Observational Studies in Epidemiology (STROBE) guidelines were followed in reporting this study.

Sample size

This study included all isolates over the study period. Therefore, the sample size was effectively all 95 fungal isolates identified and analyzed during the study period, including 86 yeast isolates and nine yeast-like isolates. Sample size calculations were not done as we analyzed the entire dataset.

Variables and data sources

Demographic factors such as patient age and sex and clinical details such as sample collection information and sample type were identified. Species-level identification and antifungal susceptibility patterns were performed using the VITEK-2 system (bioMérieux, Marcy-l’Etoile, France). The antifungal susceptibility panel included amphotericin B (AMP B), flucytosine, fluconazole, voriconazole, caspofungin, and micafungin. Data were obtained from the laboratory information system and extracted into Microsoft Excel version 21 (Microsoft Corp., Redmond, WA, USA) for further analysis.

Ethical considerations

No direct patient contact was involved in the study, and patient confidentiality was maintained through the anonymization of all data before analysis. Institutional ethical approval was not required.

Quantitative variables

Quantitative variables including patient age, minimum inhibitory concentration (MIC) (interpreted based on the Clinical and Laboratory Standards Institute breakpoints for each antifungal agent (version 2017, M60-Ed1)), and the percentage of isolates sensitive, resistant, or intermediate to each antifungal agent were determined.

Statistical methods

Descriptive statistics, including frequency and percentage distributions, were used to summarize the demographic and clinical characteristics of patients, as well as the distribution of fungal species and their antifungal susceptibility patterns. A linear regression model was used to assess the trend in the number of yeast and yeast-like isolates over the study period, with a specific focus on changes during the COVID-19 era (2020-2021). The susceptibility of isolates to each antifungal agent was determined and presented as the percentage of isolates sensitive, resistant, or intermediate to each agent.

## Results

A total of 95 fungal isolates were identified during the study period, with nearly an equal distribution of female (48, 50.52%) and male (47, 49.47%) patients. Among these isolates, 86 (90.52%) were yeast isolates, predominantly comprising NAC species (45, 52.32%), and nine (9.47%) were yeast-like isolates, including *Cryptococcus laurentii* (six isolates) and *Trichosporon asahii* (three isolates) (Table 1).

**Table 1:** Summary of fungal isolates.

Category	Female (%)	Male (%)	Total (%)
Total number of fungal isolates	48 (50.52%)	47 (49.47%)	95 (100%)
Yeast isolates	43 (50%)	43 (50%)	86 (90.52%)
Yeast-like isolates	5 (55.56%)	4 (44.44%)	9 (9.47%)
Non-albicans *Candida *(including *Candida species* where species-level identification was not done)	21 (46.67%)	24 (53.33%)	45 (52.32%)
*Candida albicans*	22 (53.66%)	19 (46.34%)	41 (47.67%)

In our study, *C. albicans* was the most prevalent species, including 43% of the total samples, with a significant presence in urine (26 isolates), sputum (11 isolates), blood (one isolate) and pus/tissue/wound swabs (three isolates). *C. auris* was comparatively rare, at 1% of the total, detected only in urine. *Candida ciferri* and *C. tropicalis* represented 6% and 9% of the samples, respectively, with occurrences in urine, sputum, and blood. *Candida famata* and *T. asahii* were 2% and 3% of the total, isolated in urine. *Candida guilliermondii* (3%) and *Candida lusitaniae *(5%) were identified in urine and other samples. *Candida parapsilosis* and *Cryptococcus laurentii* were identified in 5% and 6%, respectively, isolated in multiple sample types, including urine and sputum. *Candida *species not specified further were found in various samples, accounting for 15% of the total. Table 2 shows a diverse range of fungal species, with *C. albicans* being the predominant one in different clinical specimens.

**Table 2:** Distribution of yeast/yeast-like isolates isolated from various clinical samples. ET = endotracheal

Species	Sample							Total (%)
	Urine	Sputum	Blood	Pus/Tissue/Wound swab	Foley’s tip/Drain tip/Suction tip/ET suction	Stool	Retroperitoneal fluid	
Candida albicans	26	11	1	3	0	0	0	41 (43%)
Candida auris	1	0	0	0	0	0	0	1 (1%)
Candida ciferri	2	2	0	2	0	0	0	6 (6%)
Candida famata	2	0	0	0	0	0	0	2 (2%)
Candida guilliermondii	3	0	0	0	0	0	0	3 (3%)
Candida lusitaniae	3	0	0	1	0	1	0	5 (5%)
Candida parapsilosis	2	0	2	1	0	0	0	5 (5%)
Candida species	8	3	0	0	2	0	1	14 (15%)
Candida tropicalis	4	2	1	1	1	0	0	9 (9%)
Cryptococcus laurentii	4	0	0	0	2	0	0	6 (6%)
Trichosporon asahii	3	0	0	0	0	0	0	3 (3%)

**Figure 1:**
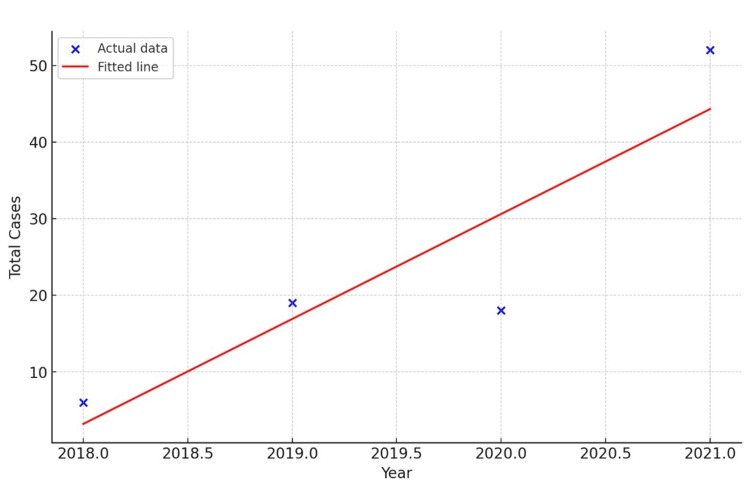
Linear regression analysis of yearly Candida cases. Blue points represent the number of cases, and the red line is the fitted linear regression model.

In the pre-COVID era (2018 and 2019), 25 (26%) isolates were identified, while in the COVID-19 era (2020 and 2021), 70 (74%) isolates were identified. A year-wise change in the trend of yeast and yeast-like isolates was observed. In the COVID-19 era (2020 and 2021), the number of cases of yeast and yeast-like infections increased. Figure 1 shows the linear regression analysis of the *Candida* cases. The blue points represent the actual data from each year, while the red line is the fitted linear regression model, showing a clear increasing trend in the number of cases over the years. Among 70 isolates in the COVID-19 era, 24% were found in samples taken from confirmed positive cases, either by antigen or real-time reverse transcription polymerase chain reaction (RT-PCR) tests.

*C. albicans* showed high sensitivity to almost all antifungal agents during the study period and no difference was observed year-wise. For AMP B, 92.68% (38 isolates) were sensitive, while 7.32% (three isolates) were resistant. For flucytosine, all isolates (100%) were sensitive. Fluconazole sensitivity was observed in 97.56% (40 isolates), with only one isolate resistant (2.44%). High sensitivity was also observed for voriconazole at 97.56% (40 isolates), caspofungin at 97.56% (40 isolates), and micafungin at 97.56% (40 isolates). *C. famata* was completely sensitive to AMP B, flucytosine, voriconazole, caspofungin, and micafungin with 100% sensitivity across these antifungals. However, for fluconazole, 50% (one isolate) was resistant and 50% (one isolate) was not determined. All *C. parapsilosis* isolates were sensitive to AMP B, flucytosine, voriconazole, caspofungin, and micafungin, while fluconazole was not tested as it is intrinsically resistant. *C. auris* exhibited 100% resistance to AMP B, while being completely sensitive (100%) to flucytosine, voriconazole, caspofungin, and micafungin. It also showed complete resistance to fluconazole. *C. ciferri* had 83.33% (five isolates) sensitivity to AMP B and 16.67% (one isolate) resistance. For flucytosine, all isolates were sensitive. For fluconazole, 66.67% (four isolates) were not determined, 16.67% (one isolate) were resistant, and 16.67% (one isolate) were sensitive. Similar mixed patterns for voriconazole, caspofungin, and micafungin were observed, with some resistance observed. The *C. guilliermondii* isolates were completely sensitive (100%) to all antifungals, including AMP B, flucytosine, fluconazole, voriconazole, caspofungin, and micafungin. *C. lusitaniae *showed 100% sensitivity to AMP B, caspofungin, and micafungin. For flucytosine, 80% (four isolates) were sensitive and 20% (one isolate) were intermediate. For fluconazole, 40% (two isolates) were sensitive, 20% (one isolate) resistant, and 40% (two isolates) were not determined. *Candida *species were highly sensitive to all antifungals, with 92.86% (13 isolates) sensitivity to AMP B, voriconazole, caspofungin, and micafungin. The fluconazole results were completely sensitive at 100% (14 isolates) with no resistance observed. *C. tropicalis *showed high sensitivity to AMP B at 88.89% (eight isolates), flucytosine at 100% (nine isolates), voriconazole at 100% (nine isolates), caspofungin at 100% (nine isolates), and micafungin at 100% (nine isolates). For fluconazole, 77.78% (seven isolates) were sensitive, and 22.22% (two isolates) were not determined. *C. laurentii* isolates were entirely sensitive (100%) to AMP B, flucytosine, fluconazole, voriconazole, caspofungin, and micafungin, with no resistance observed. The *T. asahii* isolates were also completely sensitive (100%) to AMP B, flucytosine, fluconazole, voriconazole, caspofungin, and micafungin (Table 3).

**Table 3:** Antifungal susceptibility patterns. R = resistant; S = sensitive; I = intermediate; AMP B = amphotericin B; ND = not determined

Species	AMP B (R) n (%)	AMP B (S) n (%)	Flucytosine (I) n (%)	Flucytosine (S) n (%)	Fluconazole (ND) n (%)	Fluconazole (R) n (%)	Fluconazole (S) n (%)	Voriconazole (R) n (%)	Voriconazole (S) n (%)	Caspofungin (R) n (%)	Caspofungin (S) n (%)	Micafungin (R) n (%)	Micafungin (S) n (%)
*Candida albicans*	3 (7.32%)	38 (92.68%)	0 (0.0%)	41 (100.0%)	0 (0.0%)	1 (2.44%)	40 (97.56%)	1 (2.44%)	40 (97.56%)	3 (7.32%)	40 (97.56%)	1 (2.44%)	40 (97.56%)
*Candida famata*	0 (0.0%)	2 (100.0%)	0 (0.0%)	2 (100.0%)	1 (50.0%)	1 (50.0%)	0 (0.0%)	0 (0.0%)	2 (100.0%)	0 (0.0%)	2 (100.0%)	0 (0.0%)	2 (100.0%)
*Candida parapsilosis*	0 (0.0%)	5 (100.0%)	0 (0.0%)	5 (100.0%)	5 (100.0%)	0 (0.0%)	0 (0.0%)	0 (0.0%)	5 (100.0%)	0 (0.0%)	5 (100.0%)	0 (0.0%)	5 (100.0%)
*Candida auris*	1 (100.0%)	0 (0.0%)	0 (0.0%)	1 (100.0%)	0 (0.0%)	1 (100.0%)	0 (0.0%)	0 (0.0%)	1 (100.0%)	0 (0.0%)	1 (100.0%)	0 (0.0%)	1 (100.0%)
*Candida ciferri*	1 (16.67%)	5 (83.33%)	0 (0.0%)	6 (100.0%)	4 (66.67%)	1 (16.67%)	1 (16.67%)	2 (33.33%)	4 (66.67%)	1 (16.67%)	5 (83.33%)	2 (33.33%)	4 (66.67%)
*Candida guilliermondii*	0 (0.0%)	3 (100.0%)	0 (0.0%)	3 (100.0%)	0 (0.0%)	0 (0.0%)	3 (100.0%)	0 (0.0%)	3 (100.0%)	0 (0.0%)	3 (100.0%)	0 (0.0%)	3 (100.0%)
*Candida lusitaniae*	0 (0.0%)	5 (100.0%)	1 (20.0%)	4 (80.0%)	2 (40.0%)	1 (20.0%)	2 (40.0%)	0 (0.0%)	5 (100.0%)	0 (0.0%)	5 (100.0%)	0 (0.0%)	5 (100.0%)
*Candida species*	1 (7.14%)	13 (92.86%)	0 (0.0%)	14 (100.0%)	0 (0.0%)	0 (0.0%)	14 (100.0%)	1 (7.14%)	13 (92.86%)	1 (7.14%)	13 (92.86%)	0 (0.0%)	14 (100.0%)
*Candida tropicalis*	1 (11.11%)	8 (88.89%)	0 (0.0%)	9 (100.0%)	2 (22.22%)	0 (0.0%)	7 (77.78%)	0 (0.0%)	9 (100.0%)	0 (0.0%)	9 (100.0%)	0 (0.0%)	9 (100.0%)
*Cryptococcus laurentii*	0 (0.0%)	6 (100.0%)	0 (0.0%)	6 (100.0%)	0 (0.0%)	0 (0.0%)	6 (100.0%)	0 (0.0%)	6 (100.0%)	0 (0.0%)	6 (100.0%)	0 (0.0%)	6 (100.0%)
*Trichosporon asahii*	0 (0.0%)	3 (100.0%)	0 (0.0%)	3 (100.0%)	0 (0.0%)	0 (0.0%)	3 (100.0%)	0 (0.0%)	3 (100.0%)	0 (0.0%)	3 (100.0%)	0 (0.0%)	3 (100.0%)

## Discussion

*C. albicans*,* C. glabrata*,* C. parapsilosis*,* C. tropicalis*, and *C. krusei* are the most common fungal species found on mucosal surfaces such as the skin and the respiratory, digestive, and urinary tracts. Over the last two decades, the incidence of yeast infections has increased dramatically due to increased cases of diabetes, the overuse of antibiotics, and immunosuppressive drugs such as steroids and human immunodeficiency virus infections. In the current COVID-19 era, the incidence of yeast infections has also increased [11]. Therefore, in this study, we retrospectively studied changes in the trends of isolating yeast and yeast-like species.

In our study, 86 (90.52%) of the 95 isolates were identified as yeast isolates, while nine (9.47%) were yeast-like isolates. Among yeast isolates, NAC species (52.32%) were predominant compared to *C. albicans*. A similar predominance of NAC was observed in a study by Sardi et al. [12].

*C. laurentii *(6%) emerged as a new danger, along with a few isolates of *T. asahii* (3%) in this study. Among these six *C. laurentii* isolates, four were recovered from confirmed cases of COVID-19. Surprisingly, most of the isolates were recovered from urine (four isolates), Foley’s tip (one isolate), and endotracheal tube secretions (one isolate). Only a few case reports are currently available. Londero et al. [13] reported on the relationship of *C. laurentii* with catheter-related infection, along with a change in nomenclature to *Papiliotrema laurentii*. *C.* *laurentii* is rare and commonly seen in immunosuppressed patients. Cutaneous and urinary tract infections were reported in case reports and case series by Molina-Leyva et al. [14] and Salazar-Leal et al. [15]. All *T. asahii* were recovered from urine samples taken from patients in the elderly age group. *T. asahii* with urinary tract infection was observed in a study by Khan et al. [16] in 2015 and Cronyn et al. [17] in 2021.

In this study, out of 86 yeast isolates, 41 were *C. albicans* and 45 were NAC. In 14 (31.11%) isolates, species-level identification was not performed. Among NAC in the species-level identifications, *C. tropicalis* (nine isolates), *C. ciferri* (six isolates), *C. parapsilosis* (five isolates), and *C. lusitaniae* (five isolates) emerged as common isolates. In different studies conducted throughout India and some outside India summarized in Table 4, NAC emerged as the leading cause of yeast infections, and in some studies, *C. albicans* was the most common isolate.

**Table 4:** Studies on the prevalence of yeast and yeast-like culture isolates.

Location	Study type	Prevalent isolates	Reference
Indore, Madhya Pradesh	Prospective cross-sectional	Candidiasis prevalence was 0.86%. Non-albicans *Candida* isolates (65.9%) exceeded *C. albicans* (34.07%). Notably, *C. tropicalis* was 27.4%, *C. glabrata* 16.29%, *C. krusei* 15.55%, *C. parapsilosis* 5.92%, and *C. lusitenia* 0.74%	[10]
Portugal	Retrospective analysis	A total of 906 diagnosed yeast infections, with the prominent species being *C. albicans *(69.6%), *C. glabrata* (7.6%), *C. tropicalis* (7.4%), *C. parapsilosis*, and *C. krusei*	[18]
Kurnool, India	Prospective investigation	Among 64 *Candida* isolates, the predominant species were *C. albicans* (37.5%), followed by *C. tropicalis* (32.8%), *C. krusei* (20.3%), *C. parapsilosis* (6.2%), and *C. glabrata* (3.1%)	[19]
Chennai, Tamil Nadu, India	Prospective study	A total of 250 *Candida* species were isolated, with 118 (47.2%) *C. albicans*, 64 (25.6%) *C. tropicalis*, 75 (30%) *C. glabrata*, 38 (15.2%) *C. krusei*, and 14 (5.6%) *C. parapsilosis*. Non-albicans *Candida* predominated (52.8%) over *C. albicans*	[20]
Andhra Pradesh, India	Prospective research	A total of 53 *Candida* species were isolated; 64% were identified as *C. albicans*, while 35% were non-albicans *Candida*	[21]
University of Florida, United States	Retrospective analysis	Out of 889 COVID-19 patients, 106 (12%) had candidiasis, and 14 (1.6%) specifically presented with oral candidiasis	[22]
Bhopal, Madhya Pradesh, India	Prospective cross-sectional	*C. tropicalis* (46.79%) was the predominant species, followed by* C. albicans* (37.17%) and *C. parapsilosis* (9.61%). Non-albicans *Candida* species (62.82%) outnumbered *C. albicans* (37.17%)	[23]
Silchar, Assam, India	Prospective Investigation	Out of 113 *Candida species*, 72.56% were non-albicans *Candida*, and 27.43% were *C. albicans*. Notably, *C. glabrata* constituted 32%, and *C. tropicalis* 30% of the non-albicans *Candida* isolates	[24]
Aurangabad, Maharashtra, India	Prospective Study	A total of 164 *Candida* isolates, where *C. albicans *(40.8%) was the most common species, followed by *C. tropicalis* (29.3%), *C. parapsilosis* (9.7%), *C. famata* (9.1%), *C. ciferrii* (3.6%), and *C. lusitaniae* (3%)	[25]

In this retrospective analysis, year-wise changes in the trend of isolating yeast and yeast-like isolates were observed. In the COVID-19 era (2020 and 2021), the number of cases of yeast and yeast-like infections increased. A similar incidence of yeast infections was reported in a retrospective study by Vijay et al. [26] in 2021. According to the Indian Council of Medical Research Annual Report Antimicrobial Resistance Research and Surveillance Network January 2020 to December 2020, the annual pattern of isolation indicated a consistent decrease in the isolation rate of *C. tropicalis* from 1% in 2016 to 0.76% in 2020, demonstrating a slight increase from 0.57% in 2019 to 0.76% in 2020. Simultaneously, the annual isolation rate for *C. albicans* decreased from 1% in 2016 to 0.56% in 2020. In contrast, there was an upward trajectory in the isolates of *C. auris *and *C. parapsilosis* from 2016 to 2020 [27].

Our study reports that the yearly isolation trend showed an increase in the isolation of yeast and yeast-like isolates. *C. tropicalis *(9%), *C. ciferri *(6%), *C. parapsilosis* (5%), and *C. lusitaniae *(5%) emerged as common isolates among NAC. In this study, of the 70 isolates identified during the COVID-19 era, 17 (24%) cases were positive, either by antigen or by RT-PCR test. This percentage of yeast infections is slightly higher than that reported in a retrospective analysis conducted in 2021 at the University of Florida by Katz et al. [22]. In another study, Silva et al. [28] found that most *Candida *species recovered from COVID-19 patients were isolated from the oropharynx, and *Candida *species and other yeasts were isolated from the respiratory tract in 21.4% of positive cases of COVID-19. However, in our study, out of 17 isolates of yeast and yeast-like fungi, 11 (65%) were isolated from urine samples of COVID-19-positive cases, and others were isolated from sputum, endotracheal secretion, and blood.

The limitation of this study is the lack of species-level identification for many of the NAC isolates, which may have led to an incomplete understanding of the specific types and behaviors of these fungi. Additionally, our study did not calculate morbidity and mortality rates associated with fungal infections, which limits our ability to fully assess their impact on patient health outcomes. Moreover, the absence of longitudinal follow-up restricts our understanding of the long-term effects of these infections. These gaps highlight the need for more comprehensive and detailed multicentered studies for a better understanding of fungal infection and its clinical significance and epidemiology.

## Conclusions

There was an increase in the detection of yeast and yeast-like organisms, especially NAC, during the COVID-19 era, which indicates a change in the pattern of fungal infections, probably affected by the empirical antimicrobials and steroids during the COVID-19 pandemic. The isolation of commensal fungi highlights the importance of collecting paired samples from different sites or at different times to distinguish between colonization and infection. This approach improves accuracy, guides appropriate treatment decisions, and helps prevent unnecessary antifungal use, which can lead to resistance.
